# Crossover versus Mutation: A Comparative Analysis of the Evolutionary Strategy of Genetic Algorithms Applied to Combinatorial Optimization Problems

**DOI:** 10.1155/2014/154676

**Published:** 2014-08-04

**Authors:** E. Osaba, R. Carballedo, F. Diaz, E. Onieva, I. de la Iglesia, A. Perallos

**Affiliations:** Deusto Institute of Technology (DeustoTech), University of Deusto, Avenue Universidades 24, 48007 Bilbao, Spain

## Abstract

Since their first formulation, genetic algorithms (GAs) have been one of the most widely used techniques to solve combinatorial optimization problems. The basic structure of the GAs is known by the scientific community, and thanks to their easy application and good performance, GAs are the focus of a lot of research works annually. Although throughout history there have been many studies analyzing various concepts of GAs, in the literature there are few studies that analyze objectively the influence of using blind crossover operators for combinatorial optimization problems. For this reason, in this paper a deep study on the influence of using them is conducted. The study is based on a comparison of nine techniques applied to four well-known combinatorial optimization problems. Six of the techniques are GAs with different configurations, and the remaining three are evolutionary algorithms that focus exclusively on the mutation process. Finally, to perform a reliable comparison of these results, a statistical study of them is made, performing the normal distribution *z*-test.

## 1. Introduction

Genetic algorithms (GAs) are one of the most successful metaheuristics for solving combinatorial optimization problems. Thanks to their easy application and good performance, GAs have been used to solve many complex problems framed in various fields, as, for example, transport [1, 2], software engineering [3, 4], or industry [5, 6]. GAs were proposed in 1975 by Holland [7], in an attempt to imitate the genetic process of living organisms and the law of the evolution of species. Anyway, their practical use to solve complex optimization problems was shown later, by Goldberg [8] and De Jong [9].

Throughout history, many researches have focused on the study of genetic algorithms. These studies can be grouped into 3 different categories. 

*(i) Practical Applications of GAs.* These studies focused on the application of GAs for solving specific problems. Among these three categories, this is the most common in the literature. Two subcategories can be identified in this first group of works: variations of a classic GA [10–12] or hybridization of a GA with some other technique [13–15]. 

*(ii) Development of New Operators*. These researches present new specific operators, such as crossover [16, 17] or mutation functions [18, 19]. Normally, these operators are heuristic, and they are applied to a particular problem, in which they get a great performance. 

*(iii) Analysis of the Algorithm Behavior.* These works focus on the theoretical and practical analysis of GAs. This kind of research analyzes, for example, behavioural characteristics of the algorithm, as the convergence [20], or the efficiency of certain phases of the algorithm, such as crossover [21, 22] or mutation phases [23, 24], or the influence of adapting some parameters, as the crossover and mutation probability [25–27]. These works attempt to overcome the drawbacks of traditional genetic algorithms and are the source of new problem-solving techniques, such as the adaptive genetic algorithms [28, 29] or the parallel genetic algorithms [30, 31].

In this paper a deep study on the influence of using blind crossover operators in GAs for solving combinatorial optimization problems is conducted. This study is developed by means of a comparison between GAs with this kind of operators and EAs based only on mutation operators. Thus, this work could be framed into the third category. Previously, other studies in the literature have had a similar purpose, for example [32], where the authors tried to validate the hypothesis that the crossover phase of genetic algorithms is not efficient when it is applied to routing problems. In that work, the authors develop several versions of the basic GA with some blind crossover operators (e.g., order crossover (OX) [33] or modified order crossover (MOX) [34]), and they apply these techniques to the traveling salesman problem [35]. Performances of these GAs are compared with the one of an evolutionary algorithm (EA) based solely on mutations. The comparison is based on the quality of the solution and the runtime. Furthermore, the comparison also takes into account the percentage of deviation from the average values of each parameter.

On the other hand, in [22] the efficiency of six different versions of the classic GA applied to the degree constrained minimal spanning tree problem [36] is compared. Each version has its own crossover function. In that work, the only data shown for each version of the GA is the average value of the results obtained, so, the comparison is performed based only on this criterion. Moreover, the authors do not perform the comparison of the results obtained by a conventional GA and an EA. For this reason, with this study it is not possible to quantify the real influence of the crossover phase in the optimization capacity of a GA.

Together with the above studies, in the literature there are many others that are not comparable with the study presented in this paper. The main reason is that they are focused on other types of problems [21] or because they analyzed only the crossover process of a traditional GA [37–39].

The motivation of this work stems from the absence in the literature of a study that proves objectively the efficiency of using blind crossover operators in GAs for combinatorial optimization problems. Although [32] focuses on this topic, it is only applicable to routing problems, and it is only tested with one problem, the TSP. In addition, the comparison of the results done in [32] is not as deep as the one made in the present work. On the other hand, as it has been mentioned, the study presented in the abovementioned [22] is not truly conclusive to prove the real influence of the crossover process in a GA.

Therefore, the goal of this paper is to perform an objective study on the efficiency of blind crossover operators in basic GAs with respect to blind mutation operators in basic EAs. In order to reach this goal, an exhaustive comparison between different versions of genetic and evolutionary algorithms is presented. This comparison includes the following criteria: quality of the results, runtime, and convergence behavior of each of the techniques reviewed. Furthermore, to perform a reliable comparison of these results a statistical study is made. For this purpose, the normal distribution *z*-test is performed. For the experimentation, four different problems have been used: the traveling salesman problem (TSP), the capacitated vehicle routing problem (CVRP) [40], the N-queens problem (NQP), and the one-dimensional bin packing problem (BPP) [41].

The rest of the paper is structured as follows. In Section 2 the description of the experimentation is presented. In Section 3, the tests for the TSP are shown. After that, the experiments performed with the CVRP (Section 4) are displayed, followed by those conducted with the NQP and BPP (Sections 5 and 6, resp.). Finally, the work is finished with the conclusions of the study and further work (Section 7).

## 2. Description of the Experimentation

In this section a description of the experimentation is made. First, in Section 2.1, the problems used for the tests are introduced. Then, in Section 2.2, the details of the techniques developed are described, including the functions of the different steps of the algorithms. Finally, in Section 2.3 the experimentation setup is presented.

### 2.1. Description of the Problems

For this study four different combinatorial problems have been used. Two of them are optimization problems of routing, the TSP and the CVRP. In addition, to verify that the results of this study are valid for other types of problems apart from the routing ones, two constraint satisfaction problems have also been used in the experimentation, the NQP and the BPP. These problems were chosen because they are well known, and easy to implement. In addition, they are easily replicable. In this way, any researcher can perform these same tests, either to check the results or to perform them with other crossover functions or different parameters.

The first problem used is the TSP. The TSP is one of the most famous and widely studied problems throughout history in operations research and computer science. It has a great scientific interest, and it is used in a large number of studies [42–44]. This problem can be defined on a complete graph *G* = (*V*, *A*) where *V* = {*v*_1_, *v*_2_,…, *v*_*n*_} is the set of vertexes which represents the nodes of the system, and *A* = {(*v*_*i*_, *v*_*j*_) : *v*_*i*_, *v*_*j*_ ∈ *V*, *i* ≠ *j*} is the set of arcs which represents the interconnection between nodes. Each arc has an associated distance cost *d*_*ij*_. The objective of the TSP is to find a route that visits every customer once (and only once), that is, a Hamiltonian cycle in the graph *G*, and that minimizes the total distance traveled. In a formal way, the TSP can be formulated as follows [45]:

(1) $$\begin{array}{c} \text{Minimize}f\left(X\right)=\underset{i=0}{\sum }\;\underset{i\neq j,j=0}{\sum }d_{ij}x_{ij},\;\forall i,\;j\in V, \end{array}$$

(2) $$\begin{array}{c} \text{where}\;x_{ij}\in \left\{0,1\right\},\;\forall \left\{i,j\right\}\in A, \end{array}$$

(3) $$\begin{array}{c} \text{subject to constraints}\underset{i=0,i\neq j}{\sum }x_{ij}=1,\;\forall j\in V, \end{array}$$

(4) $$\begin{array}{c} \underset{j=0,i\neq j}{\sum }x_{ij}=1,\;\forall i\in V, \end{array}$$

(5) $$\begin{array}{c} \underset{i\in S,j\in S,i\neq j}{\sum }x_{ij}\geq 1,\;\forall S\subset V, \end{array}$$

where *x*_*ij*_ in (2) a binary variable is 1 if the arc(*i*, *j*) is used in the solution. Furthermore, *V* is the set of nodes of the system and *d*_*ij*_ is the distance between the nodes *i* and *j*. The objective function, (1), is the sum of all the arcs in the solution used; that is, it is the total distance of the route. Constraints (3) and (4) indicate that each node have to be visited and abandoned only once, while the formula (5) guarantees the absence of subtours and indicates that any subset of nodes *S* has to be abandoned at least 1 time. This restriction is vital, because it avoids the presence of cycles.

**Figure 1:**
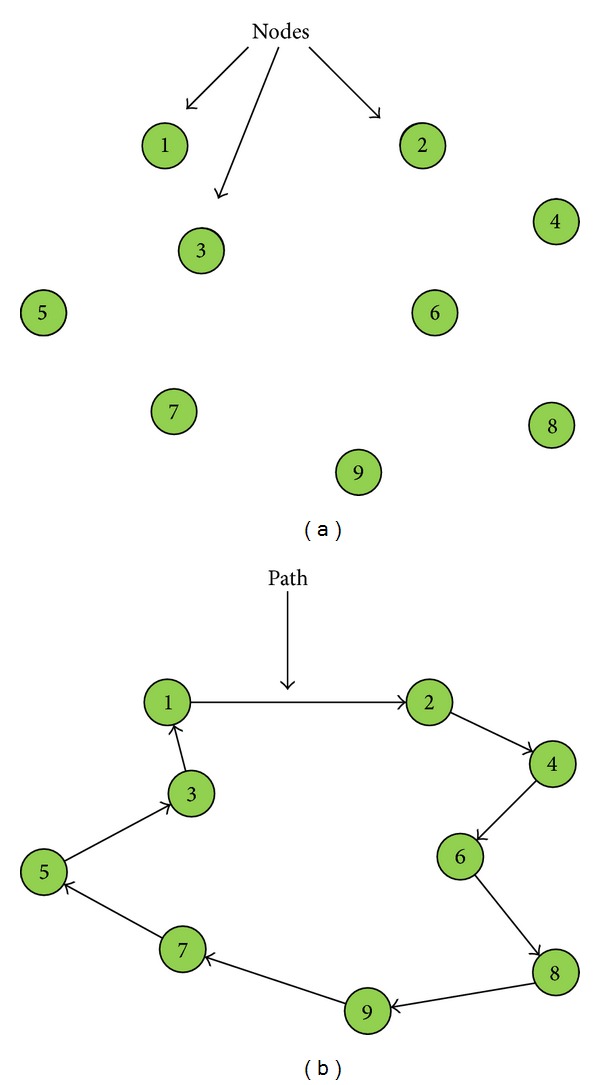
Example of TSP instance and possible solution.

Finally, all the solutions are encoded following the path representation [46]. In this way, each individual *X* is encoded by a permutation of numbers, which represents the path.  Figure 1(a) represents a possible 9-node instance of the TSP, and Figure 1(b) represents a possible solution. This solution would be encoded as *X* = (1,2, 4,6, 8,9, 7,5, 3), and its fitness would be *f*(*X*) = *d*_12_ + *d*_24_ + *d*_46_ + *d*_68_ + *d*_89_ + *d*_97_ + *d*_75_ + *d*_53_ + *d*_31_.

The second selected problem is the CVRP. Due to its complexity and, above all, its applicability to real life, the CVRP is also used in many researches every year [47, 48]. For the TSP, this problem can be defined on a complete graph. In addition, the vertex *v*_0_ represents the depot, and the rest are the customers, each of them with a demand *q*_*i*_. A fleet of vehicles *K* is available with a limited capacity *Q* for each vehicle. The objective of the CVRP is to find a number of routes with a minimum cost such that (i) each route starts and ends at the depot, (ii) each client is visited exactly by one route, and (iii) the total demand of the customers visited by one route does not exceed the total capacity of the vehicle that performs it [49]. This problem could be formulated as follows [40]:

(6) $$\begin{array}{c} \text{Minimize }\;f\left(X\right)=\underset{i=0}{\sum }\;\underset{i\neq j,j=0}{\sum }d_{ij}x_{ij}\;\forall i,j\in V, \end{array}$$

(7) $$\begin{array}{c} \text{subject to constraints}\underset{i=0,i\neq j}{\sum }x_{ij}=1,\;\forall j, \end{array}$$

(8) $$\begin{array}{c} \underset{j=0,i\neq j}{\sum }x_{ij}=1,\;\forall i\in V, \end{array}$$

(9) $$\begin{array}{c} \underset{i}{\sum }x_{ij}\geq \left|S\right|-v\left(S\right),\;\left\{S:S\subseteq \frac{V}{\left\{1\right\}},\left|S\right|\geq 2\right\}, \end{array}$$

(10) $$\begin{array}{c} \underset{i\in S}{\sum }q_{i}y_{i}^{r}\leq Q,\;\forall r\in K, \end{array}$$

(11) $$\begin{array}{c} \text{wher}\;\;y_{i}^{r}\in \left\{0,1\right\},\;\forall r\in K, \end{array}$$

(12) $$\begin{array}{c} \text{and}\;\;x_{ij}\in \left\{0,1\right\},\;\forall \left\{i,j\right\}\in A;\;i\neq j. \end{array}$$

The formula (6) is the objective function, which is the total distance traveled by all the routes. The variable (11) is a binary variable which is 1 if the vehicle *r* satisfies the demand of the client *i*, and 0 otherwise. The binary variable (12) is 1 if the arc(*i*, *j*) is used in the solution. Formulas (8) and (9) ensure that every customer is visited by one route only and exactly once. Finally, clause (9) serves to eliminate subtours, where |*S*| is the number of customers and *r*(*S*) the minimum number of vehicles to serve all. Finally, the restriction (10) ensures that the sum of all the demands of a route does not exceed the maximum vehicle capacity.

**Figure 2:**
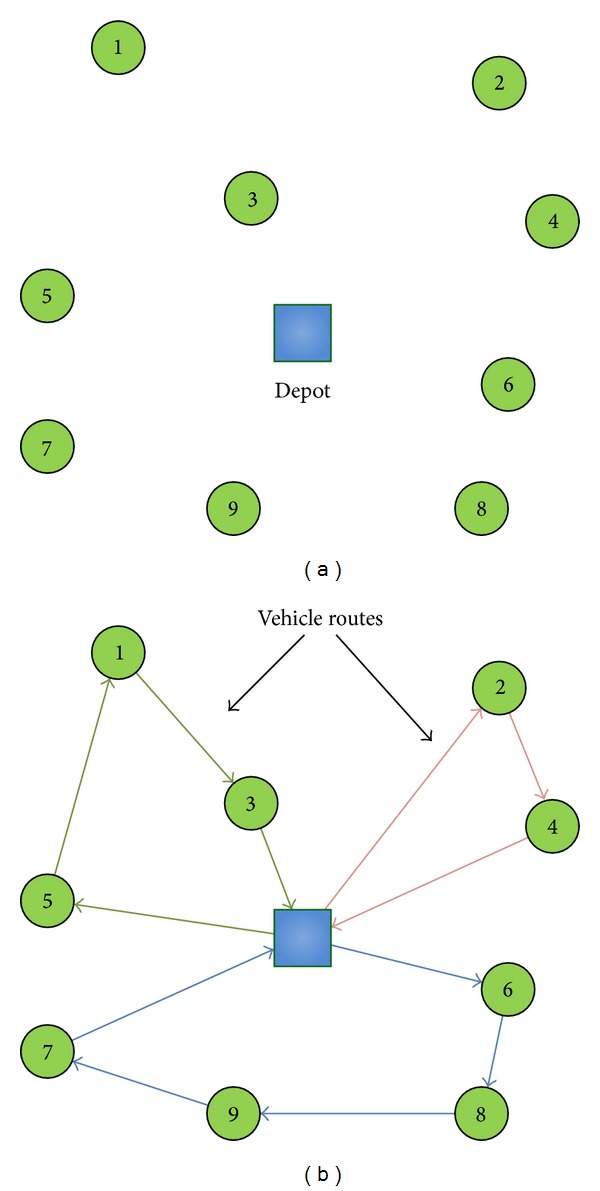
Example of CVRP instance and possible solution.

In the case of CVRP, the path representation is also used for the individuals encoding [50]. In this case, the routes are also represented as a permutation of nodes. To distinguish the routes of one solution, they are separated by zeros. In Figure 2(a) an example of a CVRP is shown. On the other hand, in Figure 2(b) a solution composed by three different routes is depicted. On this occasion, this solution would be encoded as *X* = (3,1, 5, 0, 2,4, 0, 7,9, 8,6), and its fitness would be *f*(*X*) = *d*_03_ + *d*_31_ + *d*_15_ + *d*_50_ + *d*_02_ + *d*_24_ + *d*_40_ + *d*_07_ + *d*_79_ + *d*_98_ + *d*_86_ + *d*_60_.

The third problem is the NQP. This problem is a generalization of the problem of putting eight nonattacking queens on a chessboard [51], which was introduced by Bezzel in 1848 [52]. The NQP consists of placing *N* queens on a *N* × *N* chess board, in order that they cannot attack each other; that is, on every row, column, and diagonal, only one queen can be placed. This problem is a classical combinatorial design problem (constraint satisfaction problem), which can also be formulated as a combinatorial optimization problem [53]. Although NQP is often used as benchmarking problem, it has also some real applications [54]. In this study, NQP has been formulated as a combinatorial optimization problem, where a solution *X* is coded as a *N*-tuple (*q*_1_, *q*_2_,…, *q*_*n*_), which is a permutation of the *N*-tuple (1,2,…, *N*). Each *q*_*i*_ represents the row occupied by the queen positioned in the *i*th column. Using this representation, vertical and horizontal collisions are avoided. Thus, the fitness function is defined as the number of diagonal collisions along the board. *i*th and *j*th queens collide diagonally if

(13) $$\begin{array}{c} \left|i-q_{i}\right|=\left|j-q_{j}\right|\;\forall i,j:\left\{1,2,\ldots ,N\right\};\;i\neq j. \end{array}$$

**Figure 3:**
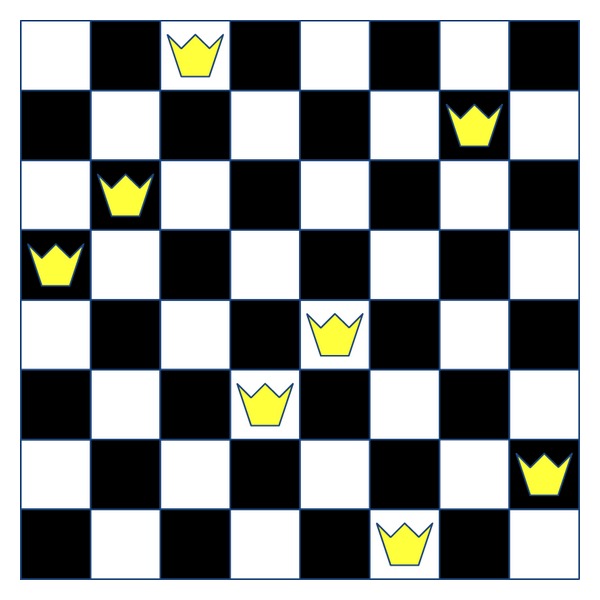
Example of a 6 × 6 instance for the NQP.

The objective is to minimize the number of conflicts, being zero the ideal fitness. An example of an individual for a 6-queens chess board could be seen in Figure 3. According to the encoding explained, the individual represented in this figure would be encoded as *X* = (2,1, 4,6, 5,3). In addition, its fitness would be 3, since there are three diagonal collisions (2-1, 1–4, and 6-5). This same formulation has been widely used in the literature [55, 56].

Finally, the last used problem is the BPP. In distribution and production, the fact of packing items into boxes or bins is a daily task. Depending on the shape and size of the items, as well as the form and capacity of bins, a wide amount of different packing problems can be formulated. The BPP is one of the simplest problems in this field [41, 57], and it is frequently used in the literature as benchmarking problem [58–60]. The BPP consists in a set of items *I* = {*i*_1_, *i*_2_,…, *i*_*n*_}, each with an associated size *s*_*i*_ and an infinite number of bins *B* of an equal capacity *q*. The objective of the BPP is to pack all the items into a minimum number of bins. Therefore, the objective function is the number of bins, which has to be minimized. In this way, given *n* items and *n* bins, the BPP can be formulated as follows:

(14) $$\begin{array}{c} \text{Minimize}\;\;f\left(X\right)=\overset{n}{\underset{i=0}{\sum }}y_{i}, \end{array}$$

(15) $$\begin{array}{c} \text{subject to constraints}\;\overset{n}{\underset{i=0}{\sum }}x_{ij}=1,\;\forall j\in \left\{1,\ldots ,n\right\}, \end{array}$$

(16) $$\begin{array}{c} \overset{n}{\underset{j=0}{\sum }}s_{i}x_{ij}\leq q,\;\forall i\in \left\{1,\ldots ,n\right\}, \end{array}$$

(17) $$\begin{array}{c} \text{where}\;\;y_{i}\in \left\{0,1\right\},\;\forall i\in \left\{1,\ldots ,n\right\}, \end{array}$$

(18) $$\begin{array}{c} \text{and}\;\;x_{ij}\in \left\{0,1\right\},\;\forall \left\{i,j\right\}\in \left\{1,\ldots ,n\right\}, \end{array}$$

where *x*_*ij*_ in (18) is a binary variable which is 1 if item *j* is put in bin *i*, and *y*_*i*_ is a variable which is 1 if bin *i* is used.

In this study, the solutions of this problem are encoded as a permutation of items. To count the number of bins needed for one solution, the size of the items is accumulated in a variable, *sumSize*. When *sumSize* exceeds *q*, the number of bins is increased in 1, and *sum*S*ize* is restarted. For example, in a simple instance of 10 items, every item *i*_*x*_ has a *s*_*i*_ = *x* and *q* = 15. One possible solution could be *X* = {(1,3, 5)(7)(9,2, 4)(6,8)(10)}, and its fitness would be 5.

### 2.2. General Description of the Developed Techniques

For the experiments, nine different techniques have been implemented and compared. The first six techniques (GA_1_, GA_2_, GA_3_, GA_4_, GA_5_, and GA_6_) are conventional GAs with different configurations. The remaining three techniques are EAs (EA_1_, EA_2_, and EA_3_). The structure used for both GAs is represented in Algorithm 1, and it is considered the conventional one. On the other hand, the flowchart of the EAs is the same, eliminating the parent selection process and crossover phase.

The parametrization of the GAs has been made based on the concepts outlined in many previous studies [61–63]. According to these researches, the crossover is considered the main operator of genetic algorithms, while the mutation is a secondary operation. In this way, GA_1_ and GA_2_ have a crossover probability (*p*_*c*_) of 90% and a mutation probability (*p*_*m*_) of 10%. In addition, GA_3_ and GA_4_ have a *p*_*c*_ = 75% and *p*_*m*_ = 25%. Finally, GA_5_ and GA_6_ have *p*_*c*_ = 50% and *p*_*m*_ = 50%. On the other hand, all the EAs have a *p*_*c*_ = 0% and a *p*_*m*_ of 100%. For GA_1_, GA_2_, and EA_1_, an initial population composed by 50 randomly created individuals is used. Additionally, for GA_3_, GA_4_, and EA_2_, the population has 75 individuals. Finally, for GA_5_, GA_6_, and EA_3_, a population composed by 100 random created individuals is used. In relation to the parents selection criteria, the well-known binary tournament criteria has been used. Regarding the survivor function, it is 50% elitist-random (which means that half of the population is composed by the best individuals, and the remaining ones are selected at random). About the ending criteria, the execution of each technique finishes when there are *n* + ∑_*k*=1_^*n*^ *i* generations without improvements in the best solution found, where *n* is the size of the problem instance.

To perform a rigorous comparison between different techniques, it is appropriate to use neutral operators throughout the implementation of them. In other words, heuristic operators that use characteristics of the problem and optimize by themselves have to be avoided. Otherwise, by using heuristic operators, the optimization capacity of the technique is influenced by the performance of these operators, and it could not be possible to determine, objectively, which is the real efficiency of the metaheuristic. In this paper, this good practice has been followed in order to make a fair comparison.

With respect to TSP, the well-known 2-opt [64] and the insertion function (IF) [65] have been used as mutation function. The first one is a classic operator which randomly selects two arcs of the solution. Then, these edges are removed from the route, and two new arcs are created, avoiding subtours. On the other hand, the second operator selects and extracts one random node of a solution and inserts it in another random position. Regarding crossover functions, the OX [33], order based crossover (OBX) [66], MOX [34], and the half crossover (HX) [67] have been used. These same mutation and crossover functions have been used for the NQP and BPP.

The OX builds the children by choosing a subroute of one of the parents and maintaining the order of the nodes of the remaining parents. First, two cut points are randomly selected, identical for both parents, and the segments between the cut points are preserved in the children. Then, starting from the second breakpoint, the remaining nodes are inserted in the same order they appear in the other parent (starting also from the second cut point), considering that the nodes that have already been inserted have to be omitted. When the end of the string is reached, it continues through the beginning of this. An example of this type of crossover could be as follows:

(19) P1=(12345678)⟶P1=(12 ∣ 345 ∣ 678)⟶O1=(∗∗ ∣ 345 ∣ ∗∗∗)⟶O1=(87 ∣ 345 ∣ 126),P2=(24687531)⟶P2=(24 ∣ 687 ∣ 531)⟶O2=(∗∗687∗∗∗)⟶O2=(45687123).

In the OBX, some random positions are selected in a parent tour. The order of the nodes in the selected positions is imposed on the other parent. For example, considering the same parents (*P*_1_ and *P*_2_) and supposing that the second, third, and sixth positions are selected, the nodes placed in these positions have to be inserted in the same order in the corresponding offspring. In this case, in *P*_2_ these nodes are 4, 6, and 5, and they have to be inserted in the first child in this same order. The rest of the route remains in the same order and position as in *P*_1_:

(20) P1=(12345678)⟶O1=(123∗∗∗78)⟶O1=(12346578).

The other child would be the next one, considering that the nodes in the second, third, and sixth positions of *P* are 2, 3, and 6:

(21) P2=(24687531)⟶O2=(∗4∗875∗1)⟶O2=(24387561).

In the case of MOX, a random cut point is selected. This cutpoint divides each parent into two sections. The nodes placed on the left part of the cut point impose their position on the other parent. Then, the remaining nodes are inserted in the children in the same order that they appear in the other parent. An example of the working way of this crossover function could be as follows:

(22) P1=(1234 ∣ 5678)⟶O1=(∗2∗4∗6∗8)⟶O1=(72543618),P2=(2468 ∣ 7531)⟶O2=(24∗∗∗∗31)⟶O2=(24567831).

The HX is a particular case of the traditional crossover, in which the cut point is made always in the middle of the path. In this way, first, a cut is made in the central position of the parents. Then, the order of nodes placed in the left part remains in the same order in the offspring. The remaining nodes are added in the same position that they can be found in the other parent. An example of the HX could be shown as follows:

(23) P1=(1234 ∣ 5678)⟶O1=(1234∗∗∗∗)⟶O1=(12346875),P2=(2468 ∣ 7531)⟶O2=(2468∗∗∗∗)⟶O2=(24681357).

On the other hand, for CVRP, the implemented crossover functions are the short route crossover (SRX), the random route crossover (RRX), and the large route crossover (LRX). These operators are a particular case of the traditional crossover, in which the cut point is made always in the middle of the chromosome. The operation of the first of them is the following: first of all, half of the routes (the shortest ones) of one of the parents is inserted in the child. After that, the nodes already selected are removed from the other parent, and the remaining nodes are inserted in the child in the same order (taking into account the vehicle capacity). Assuming a 17-node instance (including the depot), an example could be the following:

(24) $$\begin{array}{c} P_{1}=\left(1,2,3,4,0,9,10,11,12,0,13,14,15,16,0,5,6,7,8\right),\\ P_{2}=\left(1,12,6,3,0,2,4,7,11,0,5,14,16,9,0,8,13,10,15\right). \end{array}$$

The resulting offprings could be as follows:

(25) $$\begin{array}{c} O_{1}=\left(1,2,3,4,0,9,10,11,12,0,6,7,5,14,0,16,8,13,15\right),\\ O_{2}=\left(1,12,6,3,0,2,4,7,11,0,9,10,13,14,0,15,16,5,8\right). \end{array}$$

RRX works similar to the SRX. In this case, the routes selected in the first step of the process are selected randomly, instead of choosing the best ones. Finally, in the case of LRX, the selected routes are the longest ones. Regarding the mutation functions for CVRP, the vertex insertion function (VIF) and the swapping function (SF) have been used. The first one selects one random node from one randomly chosen route of the solution. This node is extracted and inserted in another randomly selected route, respecting the capacity constraints. On the other hand, in the swapping function two nodes are selected at random from two random routes to swap their positions, respecting also the capacity constraints.

In order to make the experimentation more understandable, Table 1 summarizes the characteristics of the nine algorithms used for all the problems.

### 2.3. Experimentation Setup

In this section the common aspects in all the experimentations are introduced. To begin with, all GA_1_, GA_2_, and EA_1_ were run on an Intel Core i5 2410 laptop, with 2.30 GHz and a RAM of 4 GB. The rest of the techniques were executed on an Intel Core i7 3930 computer, with 3.20 GHz and a RAM of 16 GB. Java was used as programming language. For every problem 10 different instances have been used, and for each of them 50 runs have been executed. For each experimentation, the average results, average runtime (in seconds), and convergence behaviour of every technique are shown. In addition, the standard deviation of each of them is also shown. Furthermore, for every problem three different experimentations have been performed. In each experimentation, the performance of one EA is compared with the one of two different GAs. The three experimentations differ in the configuration of the techniques.

Additionally, in order to make a fair and rigorous comparison, the normal distribution *z*-test has been performed for all experiments. Thanks to this statistical test, it can be shown whether the differences in the results obtained by each technique are significant or not. The *z* statistic has the following form:

(26) $$\begin{array}{c} z=\frac{\bar{X_{\text{EA}}}-\bar{X_{\text{GA}}}}{\sqrt{\left(\sigma_{\text{EA}}/n_{\text{EA}}\right)+\left(\sigma_{\text{GA}}/n_{\text{GA}}\right)}}, \end{array}$$

where $\bar{X_{\text{EA}}}$ is the average of an EA, *σ*_EA_ is the standard deviation of an EA, $\bar{X_{\text{GA}}}$is the average of the other technique, *σ*_GA_is the standard deviation of the other technique, *n*_EA_ is the sample size for an EA, and *n*_GA_ is the sample size for the other technique.

The *z* value can be positive (+), neutral (∗), or negative (−). The positive value of *z* indicates that the EA is significantly better. In the opposite case, the EA obtains substantially worse solutions. If *z* is neutral, the difference is not significant. The confidence interval has been stated at 95% (*z*_0.05_ = 1.96). Besides showing the symbolic value of *z*, its numerical value is also displayed. Thus, the difference in results may be seen more easily. Finally, as it has been mentioned that the *z*-test has been performed for the results quality, runtime, and convergence behaviour.

## 3. Experimentation with the TSP

In this section the experimentation with the TSP is shown. All the instances have been picked from the well-known TSPLIB benchmark [68]. In Table 2 the results and average runtimes can be found. On the other hand, in Table 3 the convergence behaviour of each technique is displayed. For this purpose, the average number of generations needed to reach the final solution is used. In Table 4 the results of the *z*-test are shown.

Several conclusions can be drawn by analyzing the results shown. First of all, looking at Table 2 it can be seen that, for the three experimentations, all the EAs perform better than the other two techniques in all the instances. According to Table 4, in the first experimentation, these differences are significant only in two cases compared to GA_1_. On the other hand, these improvements are significant in all but one instance respect to GA_2_. In the second experimentation, the EA_2_ gets significantly better results in all the instances compared with the GA_3_ and in nine instances (out of ten) compared with GA_4_. Finally, for the last experimentation, the EA_3_ significantly outperforms GA_5_ in the 100% of the instances and in the 60% (6 out of 10) regarding GA_*v*_6. For this reason, taking into account that EAs never gets worse results than the other two alternatives in the three experiments, the following conclusion can be stated.

**Conclusion 1 .**

According to the experimentation performed, the use of blind crossover operators in genetic algorithms does not offer significant improvements in the results for the TSP.

This conclusion could be explained in the following way. The main purpose of the crossover phase is to obtain new individuals making combinations of the existing ones. Although these operations were designed for the exploitation of the solution space, several studies in the literature discuss this fact [39, 63]. On the other hand, as it has been shown in several works before [69, 70], blind crossovers between different individuals can be useful to make large jumps along the solution space. For this reason, blind crossover operators applied to the TSP contribute to increase the exploration capability of the algorithm, instead of helping the exploitation.

This way, it could be said that, for the TSP, using blind crossovers helps a broad exploration of the solution space but does not help to make an exhaustive search of promising regions. This is so because it is improbable that the resulting offspring from blind crossovers can improve their parents. In addition, this fact is accentuated when the execution is near to the convergence. To get a deeper search, the existence of a function that makes little jumps in the solution space becomes necessary. The mutation function can handle this goal, and it can also contribute to perform a broad search of promising regions [71, 72]. Thus, an EA can conduct a deep and wide search, obtaining similar (or better) results to the GAs.

Regarding the runtimes, the EAs also outperform their corresponding algorithms in all the instances and experimentations. In addition, in this case these improvements are significant in all of the cases. Besides this, the differences in the runtimes become wider as the size of the instance grows. This is particularly important in real-time applications, where the runtime is a key factor. For these reasons, the following conclusion can be deduced.

**Conclusion 2 .**

In relation to the experimentation performed, the use of blind crossover operators increases significantly the execution time of an evolutionary algorithm applied to the TSP.

This difference in runtime between the GAs and the EAs can be easily explained, in the same manner as explained in the previous works [28]: comparing the working way of the crossover and mutation operators, the former are complex operations in which two individuals combine their characteristics. On the other hand, a mutation is a small modification of a chromosome and requires considerably less time than the previous ones. Thereby, the fact that an EA substitutes the crossover phase in exchange for performing more mutations is perfectly reflected in runtime, giving a great advantage to an EA in this aspect.

Finally, if the data presented in Table 3 is analyzed; first, it can be seen that both GA_1_ and GA_2_ present a better convergence behaviour compared to EA_1_. More specifically, GA_1_ is better than EA_1_ in the 80% of the cases and GA_2_ in all but one. In addition, comparing with the EA_1_, these differences are significantly better for the GA_1_ in 60% of the instances, while in 30% they are not significant. In the remaining cases, the differences are substantially better for the EA_1_. Regarding GA_2_, these data are, respectively, 60%, 40%, and 0%. Regarding the second experimentation, GA_4_ shows a significantly better convergence behaviour than EA_2_ in the 100% of the instances. On the other hand, the GA_3_ outperforms EA_2_ in the 60% on the cases, with these differences being significant in four instances (out of 10). By the way, EA_2_ significantly outperforms GA_3_ in two instances. Finally, regarding the last experimentation, the GA_5_ and GA_6_ present a substantially better convergence in the 90% and 80% of the instances, respectively. In the remaining instances, the EA_3_ shows a nonsignificant better performance. Taken into account all these data, the following conclusion can be drawn.

**Conclusion 3 .**

Considering these tests conducted for the TSP, the algorithms that use blind crossover operators demonstrate a better convergence behaviour, needing less generations to find their final solution.

This improvement in the convergence behaviour can be explained as follows. As mentioned above, blind crossover operators can be a great help to make a broad exploration of the solution space. Comparing with the mutation functions, a blind crossover can make more sudden jumps in the solution space. On the other hand, mutations are simple operations which move along the solution space little by little, conducting small jumps. For this reason and depending on the problem complexity, with the crossover functions a broader and faster exploration can be made, and the final solution can be found in less generations.

Furthermore, as has been mentioned above, mutations are an excellent option to explore the solution space. In addition, as can be seen in the results shown in Table 2, mutations can also take care of the exploitation capacity of the technique. So, using them, similar (or better) solutions can be found.

In conclusion, all the GAs converge faster than their corresponding EA. Thus, comparing with the EAs, all the versions of the GA need less generations to reach the final solution. Anyway, this fact does not mean a better performance. As can be seen in the results presented, the EAs obtain similar or significantly better results for all the TSP instances (needing a substantially smaller runtime).

## 4. Experimentation with the CVRP

In this section the experimentation with the CVRP is displayed. In this case, instances have been picked from the CVRP set of Christofides and Eilon (http://neo.lcc.uma.es/vrp (Last update: January 2013)). In Table 5 the results and average runtime can be found. Moreover, the convergence behaviour is shown in Table 6. Finally, Table 7 displays the statistical *z*-test performed for the CVRP.

The conclusions that can be drawn looking at these tables are similar to those mentioned in the previous section. In this case, regarding the quality of the results, and according to the data shown in Table 5, EA_1_ outperforms GA_1_ in 80% of the instances and GA_2_ in all of them. In addition, looking at Table 7 these improvements are significant in the 60% of the cases compared to GA_1_. On the other hand, 30% the differences are not significant, and in the remaining ones EA_1_ gets substantially worse results. Regarding GA_2_, these percentages are, respectively, 90%, 10%, and 0%.

Furthermore, EA_2_ performs better than GA_3_ in the 90% of the instances and GA_4_ in the 80%. In the case of GA_3_, the EA_2_ obtains significantly better results in nine instances. In the remaining instance, GA_3_ outperforms EA_2_ but not substantially. Moreover, EA_2_ improves significantly GA_4_ in the 50% of the instances. In addition, in the 40% these improvements are not substantially. Additionally, in the remaining instances, EA_2_ gets significantly worse results.

Finally, regarding the third experimentation, EA_3_ outperforms GA_5_ and GA_6_ in 80% of the cases. In addition, these improvements are significant in the 60% of the instances regarding both versions of the GAs. On the other hand, EA_3_ gets worse results in the 20% of the instances in relation to both GAs, but these differences are not substantial in any case.

With all this, the following finding can be stated.

**Conclusion 4 .**

According to the tests conducted for the CVRP, the use of blind crossover operators does not offer significant improvements in the results.

This conclusion can be explained in the same way that Conclusion 1 was explained in Section 3. Regarding the runtime, as in TSP, all the EAs need less time than their corresponding GAs in all the instances, with these improvements being significant in all of the cases for the first two experimentations. In the third experimentation, the differences are substantial in the 90% of the instances. In addition, as in the previous problem, these differences become higher as the size of the instance grows. For this reason, the following conclusion can be deduced.

**Conclusion 5 .**

In the same way as with the TSP, the use of crossover phase for the CVRP increases significantly the execution time of an evolutionary algorithm.

The reasons of this increase in the runtime are the same as those explained in the previous section for the TSP. Anyway, regarding the convergence behaviour, the results displayed in Table 6 are different in relation to the previously studied problem. Analyzing these outcomes it can be observed how the EAs show better convergence behaviour in all the instances and experimentations. Additionally, these improvements are significant in 80% of the cases compared to GA_2_ and GA_3_, in 70% regarding GA_2_, GA_4_, and GA_6_, and in 60% compared to GA_5_. This means that the EAs reach the final solution in less generations than the other alternatives. The following finding can be extracted from these observations.

**Conclusion 6 .**

Contrary to what happens for the TSP and according to the experimentation conducted, the use of blind crossover operators does not improve the convergence behaviour of an evolutionary algorithm applied to the CVRP.

This change in the behavior of the EA compared to that observed for the previous problems can be justified as follows. Crossover operators are complex functions that combine the characteristics of two individuals of the population. These functions are easy to design and implement if the problem has not many constraints (e.g., TSP and NQP). Anyway, if the problem has a complex formulation or its restrictions are numerous, the development of a crossover function can be very hard. For this reason, many operators designed for this type of problems include problem dependent heuristics [73, 74], or they do not consider some of the constraints of the problem [75, 76]. In any case, these operators are difficult to implement and understand, and they increase considerably the complexity of the algorithm and its runtime.

Thus, blind operators are rarely used in solving these complex problems. In addition, their performance is usually not good. An evidence of this last statement is shown in this study: all GA techniques that prioritize the use of blind crossover operators are outperformed by the technique that gives more importance to the mutation phase, in terms of exploration and exploitation.

## 5. Experimentation with the NQP

In this section the experimentation with the NQP is detailed. The characteristics of the nine techniques implemented are the same as the algorithms used for the TSP. In Table 8 the results and average runtime can be found. The name of each instance describes the number of queens and the size of the chessboard. In this case, the optimum of each instance is not shown, since it is known that it is 0 for all of them. In addition, Table 9 displays the convergence behaviour of each algorithm. On the other hand, the *z*-test made for this problems is shown in Table 10.

The conclusions that can be drawn analyzing these tables are similar to those obtained in previous sections. First of all, as can be seen in Table 8, the EAs obtain better results than their corresponding GAs in all but one of the instances. In the remaining case (8-queens instance), they get the same outcomes. In addition, these improvements are significant in 90% of the instances compared to GA_1_, GA_2_, GA_3_, GA_4_, and GA_6_, with the 8-queens instance being the only where the differences are not significant. Additionally, these improvements are substantial in the 80% of the cases regarding GA_5_, being not significant in the remaining 20%. For these reasons, Conclusions 1 and 4 are also applicable for the NQP.

The same happens with runtime. The EAs are never overcomed by any of the genetic algorithms used, obtaining significantly better runtimes in 90% and 60% of cases regarding GA_1_ and GA_2_, in 80% of the instances compared to GA_3_ and GA_4_, and in 60% and 80% in relation to GA_5_ and GA_6_, respectively. Therefore, Conclusions 2 and 5 are also applicable for this problem.

Finally, regarding the convergence behaviour, the results obtained are more similar to those seen for the TSP. Looking at the data displayed in Table 8, the EA_1_ has a better convergence behaviour in 40% of the instances and the GA_1_ and GA_2_ in the other 60%. According to Table 10, comparing to GA_1_, the differences in the results are significantly better for the EA_1_ in 20% of the instances and significantly worse in 30% of them. In the remaining cases the differences are not substantial. On the other hand, comparing to GA_2_, these percentages are, respectively, 30%, 20%, and 50%.

Regarding the second experimentation, the EA_2_ gets a better convergence compared to GA_3_ and GA_4_ in the 40% of the instances. In the remaining 60%, the EA_2_ has been overcomed by at least one of the GAs. Regarding the GA_3_, the differences are not significant in the 60% of the cases. In addition, the EA_2_ has showed a substantial better convergence behaviour in 30% of the instances. In the remaining 10% the GA_3_ has significantly outperformed the behaviour of EA_2_. On the other hand, compared to GA_4_, these percentages are different, being 50%, 10%, and 40%, respectively.

In relation to the third experimentation, the EA_3_ has shown a better convergence than GA_5_ and GA_6_ in the 20% of the cases, being overcomed in the remaining 80%. Compared to GA_5_, the difference in the behaviour is not significant in the 70% of the cases. Furthermore, they are substantially better for the GA in the remaining 30%. On the other hand, the EA_3_ has significantly improved the convergence of GA_6_ in the 10% of the instances. In addition, in the 40% of the cases the differences are not substantial. Ultimately, in the remaining 50%, GA_6_ has shown a significant better convergence behaviour.

For this reason, the following finding can be drawn.

**Conclusion 7 .**

According to the tests conducted, the use of blind crossover operators in the development of genetic algorithms for the NQP entails an improvement in the convergence behavior of the technique.

The NQP is a problem with a simple formulation. For this reason, the convergence behaviour of the GAs is much better than the one shown for the CVRP, since the crossover phase helps the exploration capacity of the technique. In this way, the results obtained in this aspect are similar to those obtained for the TSP.

## 6. Experimentation with the BPP

In this section the experimentation with the BPP is shown. The characteristics of the nine techniques developed are the same as the ones used for the TSP. In Table 11 the results and average runtime can be found. Each instance has been picked from the Scholl/Klein benchmark (http://www.wiwi.uni-jena.de/entscheidung/binpp/index.htm.). These cases are named *NxCyWz*_*a*, where *x* is 2 (100 items), 3 (200 items), or 4 (500 items); *y* is 1 (capacity of 100), 2 (capacity of 120), and 3 (capacity of 150); *z* is 1 (items size between 1 and 100) and 2 (items size between 20 and 100); *a* is A or B as benchmark indexing parameter. Additionally, Table 12 shows the convergence behaviour of each technique. Furthermore, the *z*-test made for the BPP is shown in Table 13.

The conclusions that can be obtained in this case are very similar to those drawn for the NQP. As can be seen in Table 11, the EAs obtain better or same (in two cases only) results in the 100% of the instances, being significantly better in the 90% of the cases. Therefore, Conclusions 1 and 4 can be also applied for this problem. Regarding runtimes, as already seen in the previous experimentations, all the EAs outperform their corresponding GAs. In this case, the EAs obtain significantly better runtimes in the 100% of the instances. In this way, Conclusions 2 and 5 are also valid for the BPP.

Concerning the convergence behavior, the results obtained are similar to those obtained for the NQP. The EAs have a better convergence in the 43.33% cases (13 out of 30), while the GAs perform better in the remaining 56.67%. In addition, comparing to GA_1_, the differences are significantly better for the EA_1_ in 10% (1 out of 10) of the cases and significantly worse in 20% (2 out of 10). In the remaining 7 instances these differences are insignificant. Furthermore, regarding GA_2_, these percentages are 30%, 0%, and 70%, respectively. In relation to the second experimentation, the EA_2_ shows a substantial better behaviour in 10% of the instances and substantially worse behaviour in 25%. In the rest of the instances, the differences are not substantial. Finally, for the third experimentation these percentages are, respectively, 20%, 40%, and 40%. Thereby, looking at Table 13 it can be said that Conclusion 7 is also applicable for the BPP.

## 7. Conclusions and Further Work

In this paper a study on the influence of using blind crossover operators in genetic algorithms applied to combinatorial optimization problem has been conducted. For this purpose, four different well-known combinatorial optimization problems have been used, the traveling salesman problem (TSP), the capacitated vehicle routing problem (CVRP), the N-queens problems (NQP), and the one-dimensional bin packing problem (BPP). For each problem, 10 different instances have been selected, making a total set of 40 cases. In the experimentation done, the performance of six classic genetic algorithms, each with a different crossover function, has been compared with the one of the three evolutionary algorithms.

In general, regarding the results, the EAs obtain better results in 94.16% of the cases (113 out of 120). In addition, comparing with the GA variants, these improvements are significant in the 81.25% of the cases (195 out of 240). In 17.91% of the cases (43 out of 240) these differences are insignificant, and in the remaining 0.84% (2 out of 240) one GA obtains substantially better results than its corresponding EA. For these reasons, we have the following.

**Conclusion 8 .**

Regarding the results and applicability to the experimentation performed, it is concluded that the use of blind crossover operators in genetic algorithms for solving combinatorial optimization problems provides no significant improvement in the results.

In relation to the runtime, the EAs need less time than their corresponding GAs in all of the instances. In addition, these improvements are substantial in 92.91% of the cases (223 out of 240). These data suggest the following finding.

**Conclusion 9 .**

In relation to runtime and according to the experimentation performed, the use of blind crossover operators in genetic algorithms substantially increases the execution time of the technique, without providing an improvement in results.

Regarding the convergence behaviour, the GAs show better performance than the EAs. This means that they need less generations/iterations to find their final solution. Anyway, this fact does not entail better results, or less runtime, as has been mentioned in Conclusions 8 and 9. What it really involves is a greater exploration capacity of the technique. Additionally, this fact is subject to the problem, that is, being treated and being more effective if the problem has an easy formulation. For the experimentation conducted, the EAs show better convergence behaviour in 45.83% of the cases (55 out of 120). Moreover, the statistical test conducted shows that, for simple formulation problems (TSP, NQP, and BPP), the EAs have a significantly better convergence in 12.77% (23 of 180) of the cases. On the other hand, in 41.66% (75 out of 180) of the comparisons, the GAs are substantially better. In the remaining 45.57% the differences are not remarkable. For the CVRP, as has been seen in Section 4, the EAs show a significantly better convergence in the 71.66% (43 out of 60) of the cases. As a result of this, the following finding can be deduced.

**Conclusion 10 .**

Finally, regarding the convergence behaviour and according to the experimentation performed, the study concludes that the use of blind crossover operators in genetic algorithms for solving combinatorial optimization problems with simple formulation entails a better convergence behaviour of the technique, needing less generations to obtain the final solution. Anyway, this fact does not mean better results. On the other hand, for more complex problems, the use of blind crossover operators does not imply a better convergence behavior.

Finally, as a final conclusion of this work and based on the findings that have been proposed along the paper, the following assertion can be concluded.

**Conclusion 11 .**

Based on the experimentation performed, an evolutionary algorithm (based only on mutation and survivor selection functions) is more efficient than a classic genetic algorithm to solve combinatorial optimization problems.

As a final comment, the authors of this study want to clarify that they are aware that there is a large amount of combinatorial optimization problems in the literature. In the same way, there are a lot of blind crossover operators. For these reasons, it could be pretentious to generalize the conclusions of this study to all the combinatorial optimization problems. In this work, to perform the tests, four well-known and widely used problems have been used. The goal of this selection is to choose problems of different types and to obtain conclusions as objective as possible. Following the same philosophy, all the crossover operators that have been used in this study have been previously applied in many studies in the literature. Thereby, the authors of this study are aware that the conclusions drawn are objective and rigorous, but just for the conducted experimentation.

As future work and in order to verify the conclusions of this study, it could be interesting to extend this work to some other combinatorial optimization problems, such as the minimum spanning tree problem [77] or the job-shop scheduling problem [78]. Furthermore, it may be worthwhile to investigate whether these same findings are also applicable to other types of optimization problems, such as continuous optimization.

## Figures and Tables

**Algorithm 1:**
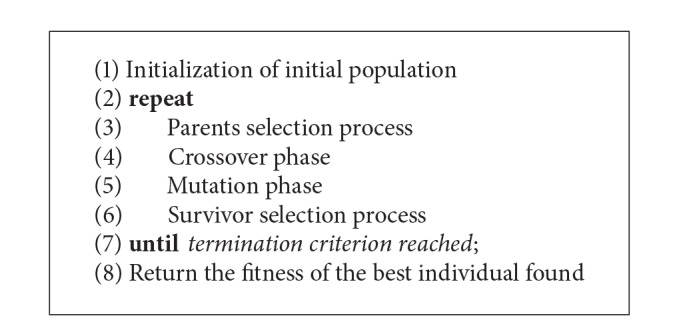
Pseudocode of all the GAs.

**Table 1:** Summary of the characteristics of all the techniques developed.

Alg.	Pop.	*p*_*c*_	*p*_*m*_	Crossover function (TSP, BPP, NQP)	Mutation function (TSP, BPP, NQP)	Crossover function (CVRP)	Mutation function (CVRP)
GA_1_	50	90%	10%	OX	2-opt	SRX	VIF
GA_2_	50	90%	10%	OBX	2-opt	RRX	VIF
EA_1_	50	0%	100%	No cross.	2-opt	No cross.	VIF

GA_3_	75	75%	25%	HX	IF	LRX	SF
GA_4_	75	75%	25%	MOX	IF	SRX	SF
EA_2_	75	0%	100%	No cross.	IF	No cross.	SF

GA_5_	100	50%	50%	OBX	2-opt	RRX	VIF
GA_6_	100	50%	50%	OX	2-opt	LRX	VIF
EA_3_	100	0%	100%	No cross.	2-opt	No cross.	VIF

**Table 2:** Results and runtimes of the nine techniques applied to the TSP. For each instance, the results, average runtime, and their standard deviations are shown.

TSP		GA_1_				GA_2_				EA_1_			
Instance		Results		Time (s)		Results		Time (s)		Results		Time (s)	
Instance	Optimum	Avg.	St.d.	Avg.	St.d.	Avg.	St.d.	Avg.	St.d.	Avg.	St.d.	Avg.	St.d.
St70	675	711.1	25.5	8.9	2.3	714.4	18.3	4.6	1.3	**705.0**	13.9	**1.5**	0.5
Eilon75	535	574.7	11.8	12.2	3.1	580.9	14.0	7.5	2.2	**570.1**	10.9	**2.2**	0.6
Eil76	538	575.6	11.4	13.0	3.0	586.1	13.1	6.7	1.9	**574.0**	11.2	**2.0**	0.7
KroA100	21282	22129.5	557.5	22.5	6.0	22376.5	546.5	14.3	4.3	**22117.0**	454.2	**3.9**	0.8
KroB100	22140	23133.2	561.3	24.5	5.6	23332.5	420.9	13.1	4.0	**23098.1**	401.6	**4.0**	0.8
KroC100	20749	21822.5	706.7	21.1	3.4	21924.4	479.2	15.4	4.4	**21642.5**	544.7	**4.1**	1.2
KroD100	21294	22347.9	573.2	24.4	7.5	22550.2	463.5	15.9	5.2	**22239.8**	383.4	**4.2**	1.0
Eil101	629	680.1	11.3	42.6	9.9	685.8	13.2	22.8	6.5	**680.0**	9.2	**4.4**	0.9
Pr107	44303	46282.2	1528.9	36.4	13.7	46470.5	1401.2	23.1	7.9	**45587.8**	936.4	**5.8**	1.8
Pr124	59030	60407.6	722.2	47.0	11.0	60678.3	1170.1	26.4	6.5	**60384.6**	927.8	**7.5**	1.2

Instance		GA_3_				GA_4_				EA_2_			

St70	675	744.6	21.9	3.6	1.1	725.2	20.5	3.4	0.8	**713.0**	10.9	**0.5**	0.1
Eilon75	535	604.2	26.6	4.6	1.1	603.9	16.5	4.9	1.2	**579.6**	14.1	**0.7**	0.1
Eil76	538	619.5	19.9	4.8	0.9	597.5	26.1	5.4	1.1	**583.7**	8.5	**0.7**	0.1
KroA100	21282	22416.4	518.4	13.3	3.0	22375.6	533.5	10.0	2.6	**22202.0**	539.4	**1.7**	0.3
KroB100	22140	23425.4	421.7	11.8	2.6	23542.6	612.1	10.1	1.9	**23024.2**	458.9	**1.7**	0.3
KroC100	20749	22304.0	634.9	11.8	2.5	22302.1	733.7	10.2	3.3	**21539.1**	468.3	**1.8**	0.3
KroD100	21294	22592.3	434.4	13.0	2.6	22797.8	629.6	8.9	2.1	**22370.8**	525.3	**1.6**	0.2
Eil101	629	718.9	17.6	16.4	3.7	712.7	15.3	17.7	3.9	**687.4**	11.1	**1.6**	0.3
Pr107	44303	46810.9	1100.7	17.1	3.1	46661.2	1242.7	13.2	3.4	**45319.4**	694.5	**2.4**	0.5
Pr124	59030	61421.5	1500.9	27.1	6.1	61148.1	1286.2	18.0	3.0	**60380.6**	669.8	**3.6**	0.5

Instance		GA_5_				GA_6_				EA_3_			

St70	675	716.1	19.8	2.8	0.6	712.4	11.6	4.9	1.0	**705.3**	10.3	**1.3**	0.2
Eilon75	535	582.8	11.9	4.0	1.1	576.2	9.9	7.7	1.5	**569.0**	7.4	**1.9**	0.6
Eil76	538	582.0	12.9	4.0	1.2	576.5	13.4	8.5	2.7	**572.7**	10.3	**1.8**	0.3
KroA100	21282	22366.4	522.9	5.5	1.5	22279.4	614.0	13.9	5.7	**21838.6**	294.3	**3.6**	0.6
KroB100	22140	23123.7	371.7	7.0	2.8	23134.9	375.8	12.7	3.9	**22964.2**	529.3	**3.6**	0.5
KroC100	20749	22005.9	584.2	6.0	1.8	21718.2	456.8	10.7	2.4	**21468.0**	400.8	**3.8**	0.8
KroD100	21294	22404.3	317.7	7.7	2.6	22163.7	356.9	13.3	4.2	**22039.1**	441.6	**3.5**	0.5
Eil101	629	696.9	16.3	11.4	2.1	689.5	12.1	24.6	5.1	**675.3**	9.8	**4.3**	0.6
Pr107	44303	46276.0	1153.6	10.5	4.2	45542.7	1053.5	25.5	9.4	**45145.4**	590.4	**4.9**	1.2
Pr124	59030	60450.1	675.1	13.7	3.2	60020.6	564.1	26.7	8.3	**59962.0**	740.7	**6.9**	0.9

**Table 3:** Convergence behaviour of the nine techniques applied to the TSP.

TSP	GA_1_		GA_2_		EA_1_	
Instance	Avg.	St.d.	Avg.	St.d.	Avg.	St.d.
St70	6093.1	1530.5	**5590.7**	2192.0	6162.6	1530.5
Eilon75	7920.3	2715.6	**7279.4**	2761.5	8439.5	5852.9
Eil76	8248.8	2663.1	**6635.9**	2481.2	7461.0	1900.5
KroA100	**9568.5**	3549.2	9980.5	3831.0	12345.7	2404.4
KroB100	10419.9	3158.8	**10090.6**	3655.4	13775.1	3594.4
KroC100	**9224.9**	3853.9	9686.7	3364.6	13614.0	3623.4
KroD100	**9495.2**	3736.6	9901.1	3919.0	13086.0	3855.8
Eil101	18646.2	5144.4	15209.5	5494.0	**15003.4**	3936.2
Pr107	13115.3	6858.9	**12489.0**	5737.5	18683.9	6795.6
Pr124	13662.3	4851.2	**11033.7**	4303.2	18917.7	4239.8

Instance	GA_3_		GA_4_		EA_2_	

St70	4400.2	1446.8	**2431.6**	770.6	3895.7	677.8
Eilon75	4868.1	1342.0	**3123.5**	940.5	4575.0	4712.5
Eil76	4954.5	1098.8	**3352.6**	922.7	4712.5	1269.3
KroA100	8382.2	2206.6	**7714.8**	3258.0	8682.8	2441.2
KroB100	7341.8	1863.3	**6516.6**	2593.8	9087.0	1879.9
KroC100	8304.0	1034.9	**3100.2**	1299.2	9824.8	1881.3
KroD100	8183.0	1886.2	**7005.9**	2796.8	8798.4	1485.5
Eil101	10241.1	2563.7	**7260.9**	1876.6	8744.9	2062.3
Pr107	8986.3	2021.3	**4540.6**	1455.5	12741.4	3343.7
Pr124	11880.6	2304.7	**9462.5**	3687.2	15258.5	2877.1

Instance	GA_5_		GA_6_		EA_3_	

St70	**4188.5**	1503.2	4748.8	1500.6	6134.2	1136.0
Eilon75	**5792.6**	2290.8	7020.5	1952.7	8631.4	2727.7
Eil76	7618.1	2425.2	7637.6	3274.4	**7521.0**	2056.8
KroA100	**3316.9**	2245.0	7032.5	4883.1	11817.8	2574.5
KroB100	**5491.8 **	4151.6	5950.4	3318.7	11619.8	2321.5
KroC100	**3928.9**	2597.3	4360.2	2136.4	12817.6	3363.8
KroD100	6494.7	3838.2	**6484.9**	3566.6	11216.1	2434.8
Eil101	**10718.5**	2877.3	14500.3	5100.6	14450.1	2938.9
Pr107	**6753.0**	4931.3	12775.2	11806.8	16436.2	5161.2
Pr124	**6387.7**	3342.2	8251.1	4914.7	18022.6	3610.0

**Table 4:** *z*-test for TSP. “+” indicates that EA is better. “−” depicts that it is worse. “∗” indicates that the difference between the two algorithms is not significant (at 95% confidence level).

TSP	EA_1_ versus GA_1_			EA_1_ versus GA_2_		
Instance	Results	Convergence	Time	Results	Convergence	Time
St70	∗ (1.46)	∗ (−0.19)	+ (21.61)	+ (4.76)	∗ (−1.51)	+ (14.99)
Eilon75	+ (2.00)	∗ (−0.56)	+ (22.02)	+ (4.30)	∗ (−1.26)	+ (16.53)
Eil76	∗ (0.69)	∗ (1.70)	+ (24.85)	+ (4.93)	∗ (−1.86)	+ (16.53)
KroA100	∗ (0.12)	− (−4.58)	+ (21.72)	+ (2.58)	− (−3.69)	+ (16.48)
KroB100	∗ (0.36)	− (−4.95)	+ (25.63)	+ (2.84)	− (−5.08)	+ (15.57)
KroC100	∗ (1.42)	− (−5.86)	+ (28.95)	+ (2.74)	− (−5.61)	+ (17.57)
KroD100	∗ (1.10)	− (−4.72)	+ (18.89)	+ (3.64)	− (−4.09)	+ (15.73)
Eil101	∗ (0.05)	+ (3.97)	+ (26.94)	+ (2.56)	∗ (0.21)	+ (19.56)
Pr107	+ (2.73)	− (−4.07)	+ (15.56)	+ (3.70)	− (−4.92)	+ (14.90)
Pr124	∗ (0.13)	− (−5.76)	+ (25.15)	∗ (1.39)	− (−9.92)	+ (20.12)

Instance	EA_2_ versus GA_3_			EA_2_ versus GA_4_		

St70	+ (9.13)	+ (2.23)	+ (25.43)	+ (3.71)	− (−10.08)	+ (19.84)
Eilon75	+ (5.77)	∗ (0.42)	+ (24.66)	+ (7.91)	− (−2.13)	+ (24.96)
Eil76	+ (11.61)	∗ (1.01)	+ (30.08)	+ (3.55)	− (−6.12)	+ (32.01)
KroA100	+ (2.02)	∗ (−0.64)	+ (22.42)	∗ (1.61)	∗ (−1.68)	+ (27.20)
KroB100	+ (4.55)	− (−4.66)	+ (30.87)	+ (4.55)	− (−5.60)	+ (27.28)
KroC100	+ (6.85)	− (−5.00)	+ (17.92)	+ (6.19)	− (−20.79)	+ (28.08)
KroD100	+ (2.29)	∗ (−1.81)	+ (24.46)	+ (3.68)	− (−4.00)	+ (30.91)
Eil101	+ (10.70)	+ (3.10)	+ (29.10)	+ (9.46)	− (−3.89)	+ (28.19)
Pr107	+ (8.10)	− (−6.76)	+ (22.22)	+ (6.66)	− (−15.90)	+ (33.10)
Pr124	+ (4.47)	− (−6.47)	+ (33.47)	+ (3.74)	− (−8.76)	+ (27.14)

Instance	EA_3_ versus GA_5_			EA_3_ versus GA_5_		

St70	+ (3.42)	− (−7.30)	+ (16.77)	+ (3.23)	− (−5.20)	+ (24.96)
Eilon75	+ (6.96)	− (−5.63)	+ (11.85)	+ (4.11)	− (−3.39)	+ (25.38)
Eil76	+ (3.98)	∗ (0.21)	+ (12.57)	∗ (1.58)	∗ (0.21)	+ (17.43)
KroA100	+ (6.21)	− (−17.59)	+ (8.31)	+ (4.57)	− (−6.12)	+ (12.7)
KroB100	+ (1.74)	− (−9.10)	+ (8.45)	∗ (1.85)	− (−9.89)	+ (16.36)
KroC100	+ (5.36)	− (−14.78)	+ (7.89)	+ (2.91)	− (−15.00)	+ (19.28)
KroD100	+ (4.74)	− (−7.34)	+ (11.21)	∗ (1.55)	− (−7.74)	+ (16.38)
Eil101	+ (8.03)	− (−6.41)	+ (22.98)	+ (6.44)	∗ (0.06)	+ (27.85)
Pr107	+ (6.16)	− (−9.59)	+ (9.06)	+ (2.32)	− (−2.00)	+ (15.37)
Pr124	+ (3.44)	− (−16.72)	+ (14.46)	∗ (0.44)	− (−11.33)	+ (16.77)

**Table 5:** Results and runtime of the nine techniques applied to the CVRP. For each instance, the results, average runtime, and their standard deviations are shown.

CVRP		GA_1_				GA_2_				EA_1_			
Instance		Results		Time (s)		Results		Time (s)		Results		Time (s)	
Instance	Optimum	Avg.	St.d.	Avg.	St.d.	Avg.	St.d.	Avg.	St.d.	Avg.	St.d.	Avg.	St.d.
En22k4	375	**389.0**	9.8	1.8	0.5	410.9	23.2	2.5	1.1	404.8	19.5	**1.1**	0.3
En23k3	569	622.7	28.9	2.1	0.9	629.9	41.6	2.3	1.0	**602.7**	30.8	**1.6**	0.6
En30k3	534	559.6	29.2	3.9	1.2	582.7	43.3	5.0	2.0	**545.8**	41.6	**2.0**	0.7
En33k4	835	**907.4**	31.9	6.0	1.8	932.8	30.6	7.0	2.3	911.9	24.9	**2.2**	0.7
En51k5	521	641.0	38.3	13.8	5.4	694.3	53.4	18.2	7.9	**628.4**	37.4	**4.5**	1.4
En76k7	682	850.0	45.7	44.4	16.4	899.5	63.3	55.1	16.5	**822.3**	42.9	**10.0**	3.3
En76k8	735	920.6	59.3	40.9	19.1	952.2	44.6	52.3	17.5	**886.9**	37.6	**8.3**	2.8
En76k14	1021	1186.9	35.6	33.4	14.0	1219.6	47.4	38.1	12.6	1**171.0**	36.2	**6.5**	2.2
En101k8	815	1061.4	54.8	107.5	33.9	1110.9	71.6	126.3	35.3	**1016.7**	49.9	**15.7**	5.1
Pr101k14	1071	1320.0	46.5	88.1	29.6	1370.7	73.1	114.9	34.3	**1270.6**	41.4	**14.8**	4.6

Instance		GA_3_				GA_4_				EA_2_			

En22k4	375	388.0	14.8	1.6	0.4	**386.1**	10.3	2.3	0.4	392.8	13.9	**0.8**	0.1
En23k3	569	622.5	31.1	2.7	1.1	615.7	37.9	2.5	1.2	**601.8**	38.4	**0.9**	0.2
En30k3	534	608.1	58.0	3.3	1.3	557.6	18.3	4.0	1.0	**547.0**	28.9	**1.4**	0.4
En33k4	835	917.0	24.9	3.4	1.3	**901.3**	29.2	3.1	1.0	903.4	23.7	**1.2**	0.4
En51k5	521	716.0	50.1	8.6	2.8	631.7	34.3	8.5	3.2	**623.9**	31.1	**2.4**	0.9
En76k7	682	847.8	48.5	35.1	13.5	835.4	56.3	26.0	10.8	**809.6**	40.8	**4.8**	1.5
En76k8	735	914.8	54.4	32.4	13.9	895.2	37.9	24.4	7.0	**870.2**	54.4	**5.1**	1.5
En76k14	1021	1198.9	46.1	24.3	8.7	1188.8	45.1	33.8	10.5	**1167.9**	28.8	**4.5**	1.9
En101k8	815	1034.2	57.8	86.9	24.3	1021.6	72.9	67.2	26.0	**1007.0**	49.4	**8.0**	2.1
Pr101k14	1071	1309.8	51.0	75.6	16.7	1288.5	45.3	59.3	25.0	**1253.2**	36.5	**8.7**	2.4

Instance		GA_5_				GA_6_				EA_3_			

En22k4	375	400.2	29.6	1.9	0.3	411.8	31.0	1.9	0.4	**390.4**	15.0	**1.7**	0.2
En23k3	569	**604.2**	37.8	2.9	0.8	608.9	32.8	2.9	1.2	613.5	40.9	**2.1**	0.8
En30k3	534	550.6	37.9	3.4	1.3	573.5	42.0	4.0	1.8	**549.8**	36.2	**2.0**	0.9
En33k4	835	914.9	33.3	3.8	1.4	904.8	24.7	3.9	1.4	**901.2**	24.8	**1.6**	0.3
En51k5	521	655.9	43.9	7.9	3.6	668.0	52.6	9.3	3.2	**636.9**	41.2	**4.9**	1.6
En76k7	682	833.1	42.0	23.2	8.1	821.8	38.5	28.8	9.5	**815.2**	29.2	**7.7**	3.7
En76k8	735	907.3	31.9	23.0	6.2	908.0	30.3	24.3	7.3	**895.1**	29.5	**8.3**	2.5
En76k14	1021	1188.3	43.8	19.0	8.3	**1171.3**	23.9	22.6	7.6	1178.1	32.5	**7.5**	3.0
En101k8	815	**1001.4**	57.2	71.9	23.9	1031.2	53.7	59.6	17.7	1006.9	57.7	**14.1**	4.8
Pr101k14	1071	1309.8	55.3	44.3	14.0	1320.0	47.2	48.2	19.2	**1285.2**	53.9	**12.3**	4.6

**Table 6:** Convergence behaviour of the nine techniques applied to the CVRP.

CVRP	GA_1_		GA_2_		EA_1_	
Instance	Avg.	St.d.	Avg.	St.d.	Avg.	St.d.
En22k4	3020.9	2216.7	5099.3	4273.6	**2358.7**	1874.9
En23k3	6717.1	5062.1	6162.3	4303.3	**5813.9**	3771.3
En30k3	9392.5	4583.3	9204.8	4963.3	**7926.0**	4872.9
En33k4	11042.1	4743.8	11628.4	5454.5	**4614.0**	3104.7
En51k5	15848.3	6991.7	18453.5	9183.2	**10387.4**	4816.7
En76k7	31420.8	13044.2	39220.3	14444.4	**19357.7**	7601.9
En76k8	27460.1	14326.1	36647.7	14385.6	**16032.6**	7197.8
En76k14	20042.1	10435.7	23084.1	8948.0	**12133.8**	6145.9
En101k8	51525.9	17393.5	55627.8	15426.4	**25925.0**	8783.4
Pr101k14	39834.9	14442.8	47396.1	14656.6	**21276.6**	6997.8

Instance	GA_3_		GA_4_		EA_2_	

En22k4	3227.0	2286.6	2551.0	1395.4	**2352.9**	1384.5
En23k3	8341.3	4495.1	5519.0	4585.9	**4128.9**	2740.6
En30k3	7837.7	5142.3	7806.9	3114.9	**7668.0**	3385.8
En33k4	6563.4	4333.6	6919.3	3760.7	**4606.3**	3169.3
En51k5	10472.0	5002.5	14226.3	7316.2	**9727.1**	5062.7
En76k7	27919.0	12521.7	25863.9	12369.9	**19385.4**	7286.1
En76k8	26178.2	13442.7	23249.8	7909.5	**19027.0**	7234.8
En76k14	16498.5	9190.2	16464.2	7082.0	**11310.7**	6886.4
En101k8	48219.9	15013.7	42115.4	17572.3	**27595.8**	8480.1
Pr101k14	38812.3	10129.9	33882.8	16091.8	**23878.2**	7960.6

Instance	GA_5_		GA_6_		EA_3_	

En22k4	2368.5	1464.5	2175.0	1989.2	**1554.5**	1313.2
En23k3	6543.4	4060.4	7632.6	5486.8	**6300.8**	2979.3
En30k3	8121.9	4806.2	8707.9	5987.3	**7977.2**	5820.4
En33k4	7586.2	4555.1	7107.0	4242.0	**4942.9**	1917.0
En51k5	10322.0	7118.3	11619.2	5673.0	**9013.9**	4690.5
En76k7	21857.2	8862.2	23312.1	9214.4	**15688.0**	9329.0
En76k8	19507.3	6989.5	19086.4	7402.4	**14941.9**	4849.8
En76k14	12945.4	7692.4	14955.9	6730.7	**10477.0**	5971.6
En101k8	44202.0	16688.4	42967.6	11510.6	**27313.3**	9049.3
Pr101k14	23547.1	9207.9	24205.8	12040.8	**17700.1**	7241.0

**Table 7:** *z*-test for CVRP. “+” indicates that EA is better. “−” depicts that it is worse. “∗” indicates that the difference between the two algorithms is not significant (at 95% confidence level).

CVRP	EA_1_ versus GA_1_			EA_1_ versus GA_2_		
Instance	Results	Convergence	Time	Results	Convergence	Time
En22k4	− (−5.09)	∗ (1.61)	+ (7.96)	∗ (1.41)	+ (4.15)	+ (8.64)
En23k3	+ (3.35)	∗ (1.01)	+ (2.76)	+ (3.71)	∗ (0.43)	+ (3.99)
En30k3	∗ (1.91)	∗ (1.55)	+ (9.45)	+ (4.33)	∗ (1.30)	+ (9.88)
En33k4	∗ (−0.78)	+ (8.01)	+ (13.24)	+ (3.73)	+ (7.90)	+ (13.69)
En51k5	∗ (1.65)	+ (4.54)	+ (11.63)	+ (7.14)	+ (5.50)	+ (12.09)
En76k7	+ (3.12)	+ (5.64)	+ (14.47)	+ (7.14)	+ (8.60)	+ (18.90)
En76k8	+ (3.42)	+ (5.03)	+ (11.89)	+ (7.90)	+ (9.06)	+ (17.45)
En76k14	+ (2.20)	+ (4.61)	+ (13.36)	+ (5.76)	+ (7.13)	+ (17.44)
En101k8	+ (4.26)	+ (9.29)	+ (18.86)	+ (7.63)	+ (11.83)	+ (21.88)
En101k14	+ (5.60)	+ (8.17)	+ (17.26)	+ (8.42)	+ (11.37)	+ (20.43)

Instance	EA_2_ versus GA_3_			EA_2_ versus GA_4_		

En22k4	∗ (−1.67)	+ (2.31)	+ (13.71)	− (−2.73)	∗ (0.71)	+ (25.72)
En23k3	+ (2.96)	+ (5.65)	+ (11.38)	∗ (1.83)	∗ (1.83)	+ (9.29)
En30k3	+ (6.66)	∗ (0.19)	+ (9.87)	+ (2.19)	∗ (0.21)	+ (17.06)
En33k4	+ (2.80)	+ (2.57)	+ (11.43)	∗ (−0.33)	+ (3.32)	+ (12.47)
En51k5	+ (11.04)	∗ (0.74)	+ (14.90)	∗ (1.19)	+ (3.57)	+ (12.97)
En76k7	+ (4.26)	+ (4.16)	+ (15.77)	+ (2.62)	+ (3.19)	+ (13.74)
En76k8	+ (4.09)	+ (3.31)	+ (13.80)	+ (2.66)	+ (2.78)	+ (19.06)
En76k14	+ (4.03)	+ (3.19)	+ (15.72)	+ (2.76)	+ (3.68)	+ (19.41)
En101k8	+ (2.52)	+ (8.45)	+ (22.87)	∗ (1.17)	+ (5.26)	+ (16.04)
En101k14	+ (6.38)	+ (8.19)	+ (28.03)	+ (4.29)	+ (3.94)	+ (14.24)

Instance	EA_3_ versus GA_5_			EA_3_ versus GA_6_		

En22k4	+ (2.08)	+ (2.92)	+ (3.92)	+ (4.39)	∗ (1.84)	+ (3.16)
En23k3	∗ (−1.18)	∗ (0.34)	∗ (1.87)	∗ (−0.62)	∗ (1.50)	∗ (1.47)
En30k3	∗ (0.10)	∗ (0.13)	+ (6.26)	+ (3.02)	∗ (0.61)	+ (7.02)
En33k4	+ (2.33)	+ (3.78)	+ (10.86)	∗ (0.72)	+ (3.28)	+ (11.35)
En51k5	+ (2.23)	∗ (1.08)	+ (5.38)	+ (3.29)	+ (2.50)	+ (8.69)
En76k7	+ (2.47)	+ (3.39)	+ (12.30)	∗ (0.96)	+ (4.11)	+ (14.63)
En76k8	+ (1.98)	+ (3.79)	+ (15.54)	+ (2.15)	+ (3.31)	+ (14.66)
En76k14	∗ (1.32)	∗ (1.79)	+ (9.21)	∗ (−1.19)	+ (3.51)	+ (13.06)
En101k8	∗ (−0.47)	+ (6.29)	+ (16.76)	+ (2.17)	+ (7.56)	+ (17.54)
En101k14	+ (2.25)	+ (3.52)	+ (15.35)	+ (3.44)	+ (3.27)	+ (12.85)

**Table 8:** Results and runtime of the nine techniques applied to the NQP. For each instance, the results, average runtime, and their standard deviations are shown.

NQP	GA_1_				GA_2_				EA_1_			
Instance	Results		Time (s)		Results		Time (s)		Results		Time (s)	
Instance	Avg.	St.d.	Avg.	St.d.	Avg.	St.d.	Avg.	St.d.	Avg.	St.d.	Avg.	St.d.
8-queens	**0.0**	0.0	**0.1**	0.0	0.1	0.2	**0.1**	0.0	**0.0**	0.0	**0.1**	0.0
20-queens	1.6	0.8	**0.1**	0.1	1.5	0.7	**0.1**	0.1	**0.8**	0.5	**0.1**	0.0
50-queens	6.6	1.6	0.6	0.1	6.4	1.6	0.3	0.1	**5.1**	1.4	**0.3**	0.1
75-queens	13.7	2.2	0.8	0.3	13.1	2.5	0.7	0.4	**9.2**	2.3	**0.6**	0.1
100-queens	15.4	2.3	6.2	1.5	15.2	2.6	4.7	1.3	**11.5**	2.3	**2.9**	0.7
125-queens	25.5	3.4	5.2	1.5	24.3	3.6	3.9	1.2	**17.0**	3.1	**3.6**	0.8
150-queens	32.0	3.9	9.5	3.4	27.7	3.9	7.6	2.2	**21.9**	3.2	**6.6**	1.4
200-queens	43.2	5.9	69.9	7.9	38.2	4.5	38.0	8.1	**26.6**	3.9	**32.5**	7.9
250-queens	56.4	7.1	63.8	19.8	52.1	5.2	45.5	12.5	**38.0**	5.3	**42.5**	10.7
300-queens	69.9	7.9	123.3	41.3	65.2	6.5	109.5	25.6	**45.6**	5.3	**94.6**	19.3

Instance	GA_3_				GA_4_				EA_2_			

8-queens	**0.0**	0.0	**0.1**	0.1	**0.0**	0.0	**0.1**	0.1	**0.0**	0.0	**0.1**	0.1
20-queens	1.4	1.0	**0.1**	0.1	1.3	0.8	**0.1**	0.1	**0.8**	0.6	**0.1**	0.1
50-queens	5.9	1.8	0.2	0.1	5.6	1.3	0.2	0.1	**4.6**	1.5	**0.1**	0.1
75- queens	10.9	2.1	0.7	0.1	10.0	2.5	0.8	0.1	**8.7**	1.6	**0.5**	0.1
100-queens	14.7	3.3	2.2	0.6	15.3	2.8	1.8	0.5	**12.1**	2.0	**1.5**	0.3
125-queens	19.8	2.9	4.2	1.1	18.3	2.7	4.8	1.1	**17.2**	2.5	**3.1**	0.5
150-queens	23.7	3.7	8.1	2.7	22.2	3.2	9.3	2.0	**21.3**	3.0	**5.8**	1.0
200-queens	33.3	4.4	26.7	7.2	30.4	4.3	27.1	6.1	**26.9**	4.8	**18.7**	4.0
250-queens	43.5	5.6	52.6	12.0	41.6	5.2	56.4	13.1	**37.1**	4.5	**44.8**	9.1
300-queens	57.8	5.7	98.6	33.6	50.4	6.5	118.6	28.5	**45.9**	4.9	**77.6**	19.7

Instance	GA_5_				GA_6_				EA_3_			

8-queens	**0.0**	0.0	**0.1**	0.1	**0.0**	0.0	**0.1**	0.0	**0.0**	0.0	**0.1**	0.0
20-queens	1.3	0.6	**0.1**	0.1	1.1	0.5	**0.1**	0.1	**0.8**	0.6	**0.1**	0.1
50-queens	5.2	1.6	0.2	0.1	4.9	1.2	0.2	0.1	**4.2**	1.4	**0.1**	0.1
75-queens	10.0	2.0	0.9	0.1	8.7	1.9	0.8	0.1	**7.6**	2.3	**0.6**	0.1
100-queens	12.7	2.7	2.6	0.3	13.4	2.6	2.5	0.6	**11.8**	2.1	**2.1**	0.4
125-queens	17.8	2.1	6.3	0.9	15.6	3.1	5.1	1.0	**14.4**	2.7	**4.7**	1.0
150-queens	21.2	4.3	8.2	2.7	21.2	2.7	8.6	1.9	**19.5**	3.3	**7.7**	1.6
200-queens	30.3	3.5	28.6	3.9	30.5	3.8	25.8	5.3	**27.0**	4.2	**22.9**	5.0
250-queens	36.9	3.7	59.1	11.6	36.2	3.0	62.5	10.9	**32.1**	4.1	**52.8**	10.0
300-queens	46.7	7.0	93.5	21.9	46.9	4.6	111.3	27.2	**42.5**	6.6	**89.7**	16.8

**Table 9:** Convergence behaviour of the nine techniques applied to the NQP.

NQP	GA_1_		GA_2_		EA_1_	
Instance	Avg.	St.d.	Avg.	St.d.	Avg.	St.d.
8-queens	3.3	3.2	4.9	4.6	**2.8**	2.9
20-queens	**36.9**	26.2	37.9	28.3	39.1	21.8
50-queens	210.0	126.6	191.3	112.3	**151.9**	71.4
75-queens	**183.4**	98.8	195.6	93.5	224.8	89.0
100-queens	818.0	385.7	791.7	333.7	**575.8**	255.8
125-queens	589.1	202.6	599.7	217.8	**578.9**	173.9
150-queens	**636.0**	290.9	788.1	299.1	723.1	209.1
200-queens	**1181.6**	417.3	1560.7	563.6	1854.1	606.3
250-queens	**1649.7**	615.9	1717.2	564.3	1853.6	567.6
300-queens	**2279.4**	897.3	2402.7	843.5	2821.7	683.3

Instance	GA_3_		GA_4_		EA_2_	

8-queens	3.0	1.8	2.1	1.3	**1.8**	1.2
20-queens	18.5	10.3	**18.2**	8.9	23.9	10.9
50-queens	128.0	54.3	131.2	48.4	**116.0**	38.1
75-queens	207.4	82.0	**176.6**	65.7	210.7	74.3
100-queens	416.3	208.9	**252.7**	112.3	346.7	105.9
125-queens	511.7	187.0	**451.1**	151.0	462.4	105.8
150-queens	712.5	292.0	654.1	190.8	**614.1**	159.1
200-queens	1363.1	458.5	1351.0	345.4	**1208.5**	330.6
250-queens	1714.2	480.1	**1461.5**	433.2	1827.3	432.4
300-queens	2465.8	948.1	**2222.1**	636.2	2250.8	675.3

Instance	GA_5_		GA_6_		EA_3_	

8-queens	1.6	1.2	2.3	1.5	**1.4**	1.1
20-queens	21.2	9.1	21.7	6.8	**21.1**	10.8
50-queens	89.2	42.4	**80.0**	31.4	91.6	38.1
75-queens	**154.3**	65.1	159.0	55.3	172.3	65.8
100-queens	**240.8**	89.4	315.4	124.2	325.2	102.9
125-queens	**329.8**	98.8	420.3	127.1	569.5	145.9
150-queens	590.2	254.0	**484.4**	164.0	672.8	199.4
200-queens	**847.0**	237.5	903.6	250.1	1193.7	337.6
250-queens	**1344.2**	399.0	1491.5	329.7	1470.1	360.6
300-queens	1974.5	975.1	**1829.6**	567.3	2137.0	492.9

**Table 10:** *z*-test for NQP. “+” indicates that EA is better. “−” depicts that it is worse. “∗” indicates that the difference between the two algorithms is not significant (at 95% confidence level).

NQP	EA_1_ versus GA_1_			EA_1_ versus GA_2_		
Instance	Results	Convergence	Time	Results	Convergence	Time
8-queens	∗ (0.00)	∗ (0.80)	∗ (0.00)	∗ (1.41)	+ (2.70)	∗ (0.00)
20-queens	+ (5.08)	∗ (−0.57)	+ (15.00)	+ (5.07)	∗ (−0.75)	+ (6.32)
50-queens	+ (4.76)	+ (2.84)	+ (10.00)	+ (4.03)	+ (2.10)	+ (2.16)
75-queens	+ (9.60)	− (−2.19)	+ (4.14)	+ (7.88)	∗ (−1.58)	∗ (1.06)
100-queens	+ (8.23)	+ (3.69)	+ (13.04)	+ (7.37)	+ (3.63)	+ (7.82)
125-queens	+ (12.98)	∗ (0.26)	+ (6.30)	+ (10.85)	∗ (0.52)	∗ (1.10)
150-queens	+ (13.86)	∗ (1.71)	+ (5.40)	+ (13.86)	∗ (1.25)	+ (2.61)
200-queens	+ (16.48)	− (−6.45)	+ (23.56)	+ (13.64)	− (−2.50)	+ (3.39)
250-queens	+ (14.54)	∗ (−1.72)	+ (6.67)	+ (14.54)	∗ (1.20)	∗ (1.29)
300-queens	+ (18.06)	− (−3.39)	+ (4.44)	+ (16.47)	− (−2.72)	+ (3.27)

Instance	EA_2_ versus GA_3_			EA_2_ versus GA_4_		

8-queens	∗ (0.00)	+ (3.92)	∗ (0.00)	∗ (0.00)	∗ (1.19)	∗ (0.00)
20-queens	+ (3.63)	− (−2.54)	∗ (0.00)	+ (3.53)	− (−2.81)	∗ (0.00)
50-queens	+ (3.93)	∗ (1.27)	+ (5.00)	+ (3.56)	∗ (1.74)	+ (5.00)
75-queens	+ (5.89)	∗ (−0.21)	+ (10.00)	+ (3.09)	− (−2.43)	+ (15.00)
100-queens	+ (4.76)	+ (2.09)	+ (7.37)	+ (6.57)	− (−4.30)	+ (3.63)
125-queens	+ (4.80)	∗ (1.61)	+ (6.43)	+ (2.11)	∗ (−0.43)	+ (9.94)
150-queens	+ (3.56)	+ (2.09)	+ (5.64)	+ (1.45)	∗ (1.13)	+ (11.06)
200-queens	+ (6.94)	∗ (1.93)	+ (6.86)	+ (3.84)	+ (2.10)	+ (8.14)
250-queens	+ (6.29)	∗ (−1.23)	+ (3.66)	+ (4.62)	− (4.22)	+ (5.14)
300-queens	+ (11.19)	∗ (1.30)	+ (3.81)	+ (3.92)	∗ (−0.21)	+ (8.36)

Instance	EA_3_ versus GA_5_			EA_3_ versus GA_6_		

8-queens	∗ (0.00)	∗ (0.86)	∗ (0.00)	∗ (0.00)	+ (3.42)	∗ (0.00)
20-queens	+ (4.16)	∗ (0.05)	∗ (0.00)	+ (2.71)	∗ (0.33)	∗ (0.00)
50-queens	+ (3.32)	∗ (−0.29)	+ (5.00)	+ (2.68)	∗ (−1.66)	+ (5.00)
75-queens	+ (5.56)	∗ (−1.37)	+ (15.00)	+ (2.60)	∗ (−1.09)	+ (10.00)
100-queens	∗ (1.86)	− (−4.37)	+ (7.07)	+ (3.38)	− (−0.42)	+ (3.92)
125-queens	+ (7.02)	− (−9.61)	+ (8.40)	+ (2.06)	− (−5.45)	+ (2.00)
150-queens	+ (2.21)	∗ (−1.80)	∗ (1.12)	+ (2.81)	− (−5.15)	+ (2.56)
200-queens	+ (4.26)	− (−5.93)	+ (6.35)	+ (4.36)	− (−4.88)	+ (2.81)
250-queens	+ (6.14)	∗ (−1.65)	+ (2.90)	+ (5.70)	∗ (0.30)	+ (4.63)
300-queens	+ (3.06)	∗ (−1.05)	∗ (0.97)	+ (3.86)	− (−2.89)	+ (4.77)

**Table 11:** Results and runtimes of the nine techniques applied to the BPP. For each instance, the results, average runtime, and their standard deviations are shown.

BPP		GA_1_				GA_2_				EA_1_			
Instance		Results		Time (s)		Results		Time (s)		Results		Time (s)	
Instance	Optimum	Avg.	St.d.	Avg.	St.d.	Avg.	St.d.	Avg.	St.d.	Avg.	St.d.	Avg.	St.d.
N2C1W1_A	48	53.4	0.7	0.35	0.12	53.7	0.7	0.08	0.03	**53.1**	0.7	**0.02**	0.01
N2C1W1_B	49	54.3	0.7	0.29	0.08	54.4	0.8	0.09	0.02	**53.3**	0.5	**0.02**	0.01
N3C2W2_A	107	121.4	1.5	1.84	0.33	121.8	1.4	0.47	0.16	**120.2**	1.3	**0.07**	0.02
N3C2W2_B	105	117.7	1.8	1.93	0.54	118.2	2.2	0.39	0.20	**116.7**	1.1	**0.06**	0.03
N3C3W1_A	66	73.9	0.8	1.48	0.42	73.6	0.8	0.42	0.18	**73.2**	0.9	**0.07**	0.03
N3C3W1_B	71	80.4	0.9	1.46	0.37	79.8	0.7	0.46	0.24	**79.2**	0.9	**0.06**	0.02
N4C1W1_A	240	277.9	2.4	7.79	2.90	275.4	2.4	5.84	1.85	**273.4**	1.7	**0.37**	0.12
N4C1W1_B	262	300.4	3.2	7.48	3.12	298.8	1.4	5.93	2.15	**295.8**	2.2	**0.45**	0.21
N4C1W1_C	241	277.9	2.6	7.67	2.69	276.8	2.7	6.15	2.05	**273.6**	1.6	**0.49**	0.18
N4C2W1_A	210	245.8	2.9	7.08	2.41	244.8	2.1	6.02	1.99	**242.6**	1.9	**0.51**	0.24

Instance		GA_3_				GA_4_				EA_2_			

N2C1W1_A	48	53.2	0.9	0.37	0.10	53.4	0.8	0.06	0.02	**52.8**	0.6	**0.01**	0.01
N2C1W1_B	49	54.0	0.5	0.25	0.12	54.1	0.7	0.08	0.02	**53.5**	0.6	**0.01**	0.01
N3C2W2_A	107	121.0	1.3	1.93	0.41	122.0	1.5	0.51	0.19	**120.4**	1.5	**0.06**	0.02
N3C2W2_B	105	117.4	1.5	2.12	0.77	117.9	1.9	0.40	0.22	**116.8**	1.0	**0.05**	0.01
N3C3W1_A	66	74.2	1.0	1.82	0.57	73.3	0.5	0.58	0.21	**73.0**	0.6	**0.08**	0.02
N3C3W1_B	71	80.1	0.7	1.39	0.28	79.5	1.1	0.49	0.32	**78.9**	1.0	**0.06**	0.03
N4C1W1_A	240	276.3	2.7	7.91	2.49	274.3	2.1	6.12	2.09	**273.5**	1.4	**0.43**	0.26
N4C1W1_B	262	299.8	3.4	8.27	3.93	299.4	1.8	6.29	2.77	**295.3**	2.0	**0.51**	0.28
N4C1W1_C	241	278.2	2.9	8.93	3.00	277.1	2.2	7.00	2.22	**272.9**	1.9	**0.68**	0.25
N4C2W1_A	210	245.2	3.1	8.11	2.91	245.1	2.1	5.99	2.42	**242.9**	2.1	**0.89**	0.32

Instance		GA_5_				GA_6_				EA_3_			

N2C1W1_A	48	52.9	0.8	0.41	0.09	**52.7**	0.9	0.21	0.12	**52.7**	0.6	**0.02**	0.01
N2C1W1_B	49	53.8	0.7	0.31	0.12	53.5	0.7	0.32	0.11	**52.8**	0.7	**0.02**	0.01
N3C2W2_A	107	119.2	1.1	1.95	0.77	120.1	1.6	1.84	0.70	**118.9**	1.1	**0.08**	0.03
N3C2W2_B	105	117.2	1.9	1.99	0.71	117.4	2.7	1.72	0.81	**116.4**	1.4	**0.09**	0.04
N3C3W1_A	66	73.8	0.7	2.11	0.70	**72.8**	0.9	2.21	1.00	**72.8**	0.7	**0.10**	0.03
N3C3W1_B	71	80.1	1.2	2.01	0.54	78.4	1.1	1.87	0.91	**78.1**	0.5	**0.12**	0.04
N4C1W1_A	240	278.1	2.8	7.89	2.71	276.0	2.8	6.84	2.08	**273.5**	1.9	**0.47**	0.12
N4C1W1_B	262	298.4	3.7	8.21	3.03	297.1	2.1	6.94	2.72	**295.1**	2.8	**0.53**	0.32
N4C1W1_C	241	277.1	2.2	9.00	3.09	275.9	2.4	8.95	2.71	**273.0**	2.1	**0.81**	0.37
N4C2W1_A	210	242.4	3.1	8.15	3.12	244.1	2.6	7.99	2.40	**241.7**	1.5	**0.97**	0.42

**Table 12:** Convergence behaviour of the nine techniques applied to the BPP.

BPP	GA_1_		GA_2_		EA_1_	
Instance	Avg.	St.d.	Avg.	St.d.	Avg.	St.d.
N2C1W1_A	134.4	85.0	143.8	76.8	**128.7**	88.8
N2C1W1_B	**64.8**	24.6	112.8	81.4	86.9	34.7
N3C2W2_A	332.1	144.1	384.9	153.7	**301.2**	185.7
N3C2W2_B	356.4	116.7	345.1	128.0	**314.8**	111.0
N3C3W1_A	**298.7**	102.4	310.8	117.0	332.1	98.6
N3C3W1_B	**366.0**	176.8	410.2	218.4	385.8	158.4
N4C1W1_A	1542.3	312.7	1569.7	583.9	**1328.6**	586.9
N4C1W1_B	1663.4	497.8	1682.4	597.7	**1538.7**	486.8
N4C1W1_C	**1364.8**	599.4	1473.1	757.2	1499.4	584.7
N4C2W1_A	**1340.0**	573.0	1495.5	674.6	1616.4	473.5

Instance	GA_3_		GA_4_		EA_2_	

N2C1W1_A	151.7	80.8	132.2	81.2	**112.7**	90.7
N2C1W1_B	**87.4**	43.1	95.6	42.2	100.3	56.1
N3C2W2_A	**232.7**	101.8	299.4	81.4	285.7	91.3
N3C2W2_B	371.5	120.7	**301.4**	114.7	350.0	103.3
N3C3W1_A	312.8	136.9	358.7	136.2	**299.5**	77.0
N3C3W1_B	**351.7**	146.2	400.7	187.4	411.4	101.3
N4C1W1_A	1501.1	304.7	1499.0	608.9	**1482.4**	499.9
N4C1W1_B	**1452.8**	531.5	1577.3	519.0	1490.2	503.1
N4C1W1_C	1612.7	671.4	1579.0	676.3	**1535.8**	555.3
N4C2W1_A	**1315.8**	500.4	1399.4	741.2	1584.4	463.9

Instance	GA_5_		GA_6_		EA_3_	

N2C1W1_A	114.0	73.4	**100.4**	57.1	142.7	90.4
N2C1W1_B	81.4	21.1	**71.8**	27.4	95.7	43.8
N3C2W2_A	**300.2**	112.4	327.1	99.7	350.2	198.7
N3C2W2_B	376.4	132.4	355.4	140.5	**299.4**	134.5
N3C3W1_A	280.7	139.5	**273.0**	113.6	350.7	102.7
N3C3W1_B	481.8	241.5	451.9	223.4	**371.4**	188.0
N4C1W1_A	1427.0	299.9	1500.2	531.5	**1286.7**	499.7
N4C1W1_B	1701.8	513.8	1759.0	642.3	**1612.0**	500.1
N4C1W1_C	1310.8	524.3	**1210.4**	571.8	1571.0	611.4
N4C2W1_A	**1274.0**	497.9	1379.6	573.4	1527.1	511.7

**Table 13:** *z*-test for BPP. “+” indicates that EA is better. “−” depicts that it is worse. “∗” indicates that the difference between the two algorithms is not significant (at 95% confidence level).

BPP	EA_1_ versus GA_1_			EA_1_ versus GA_2_		
Instance	Results	Convergence	Time	Results	Convergence	Time
N2C1W1_A	+ (2.14)	∗ (0.32)	+ (19.37)	+ (4.28)	∗ (0.90)	+ (13.41)
N2C1W1_B	+ (8.21)	− (−3.67)	+ (23.68)	+ (8.24)	+ (2.06)	+ (22.13)
N3C2W2_A	+ (4.27)	∗ (0.92)	+ (37.85)	+ (5.92)	+ (2.45)	+ (17.54)
N3C2W2_B	+ (3.35)	∗ (1.82)	+ (24.44)	+ (5.95)	∗ (1.26)	+ (11.53)
N3C3W1_A	+ (4.11)	∗ (−1.66)	+ (23.67)	+ (2.34)	∗ (−0.98)	+ (13.56)
N3C3W1_B	+ (6.66)	∗ (−0.58)	+ (26.71)	+ (3.72)	∗ (0.63)	+ (11.74)
N4C1W1_A	+ (10.81)	+ (2.16)	+ (18.07)	+ (4.80)	+ (1.97)	+ (20.86)
N4C1W1_B	+ (8.37)	∗ (1.26)	+ (15.89)	+ (8.13)	∗ (1.31)	+ (17.93)
N4C1W1_C	+ (9.95)	∗ (−1.13)	+ (18.83)	+ (7.20)	∗ (−0.19)	+ (19.44)
N4C2W1_A	+ (6.52)	− (−2.62)	+ (19.18)	+ (5.49)	∗ (−1.03)	+ (19.43)

Instance	EA_2_ versus GA_3_			EA_2_ versus GA_4_		

N2C1W1_A	+ (2.61)	+ (2.27)	+ (25.32)	+ (4.24)	∗ (1.16)	+ (15.81)
N2C1W1_B	+ (9.05)	∗ (−1.28)	+ (14.09)	+ (8.43)	∗ (−0.33)	+ (22.13)
N3C2W2_A	+ (2.13)	− (−2.74)	+ (32.21)	+ (5.33)	∗ (0.54)	+ (16.65)
N3C2W2_B	+ (2.35)	∗ (0.95)	+ (19.00)	+ (3.62)	− (−2.08)	+ (11.23)
N3C3W1_A	+ (7.27)	∗ (0.59)	+ (21.57)	+ (2.71)	+ (2.98)	+ (16.76)
N3C3W1_B	+ (6.95)	− (−2.37)	+ (33.39)	+ (2.85)	∗ (−0.31)	+ (9.46)
N4C1W1_A	+ (6.50)	∗ (0.22)	+ (21.12)	+ (2.24)	∗ (0.15)	+ (19.10)
N4C1W1_B	+ (8.06)	∗ (−0.36)	+ (13.92)	+ (10.77)	∗ (0.78)	+ (14.67)
N4C1W1_C	+ (10.80)	∗ (0.62)	+ (19.37)	+ (10.21)	∗ (0.32)	+ (20.00)
N4C2W1_A	+ (4.34)	− (−2.78)	+ (17.43)	+ (5.23)	− (−1.97)	+ (14.77)

Instance	EA_3_ versus GA_5_			EA_3_ versus GA_6_		

N2C1W1_A	∗ (1.41)	∗ (−1.74)	+ (30.45)	∗ (0.00)	− (−2.79)	+ (11.15)
N2C1W1_B	+ (7.14)	− (−2.07)	+ (17.02)	+ (5.00)	− (−3.27)	+ (19.20)
N3C2W2_A	∗ (1.36)	∗ (−1.54)	+ (17.15)	+ (4.37)	∗ (−0.73)	+ (17.76)
N3C2W2_B	+ (2.39)	+ (2.88)	+ (18.89)	+ (2.32)	+ (2.03)	+ (14.21)
N3C3W1_A	+ (7.14)	− (−2.85)	+ (20.28)	∗ (0.00)	− (3.58)	+ (14.91)
N3C3W1_B	+ (10.87)	+ (2.55)	+ (24.68)	∗ (1.75)	∗ (1.94)	+ (13.58)
N4C1W1_A	+ (9.61)	∗ (1.70)	+ (19.34)	+ (5.22)	+ (2.06)	+ (21.61)
N4C1W1_B	+ (5.02)	∗ (0.88)	+ (17.82)	+ (4.04)	∗ (1.27)	+ (16.54)
N4C1W1_C	+ (9.53)	− (−2.28)	+ (18.60)	+ (6.43)	− (−3.01)	+ (21.04)
N4C2W1_A	∗ (1.43)	− (−2.50)	+ (16.12)	+ (5.65)	∗ (1.21)	+ (20.37)
